# Clinical use of SAND battery to evaluate language in patients with Progressive Supranuclear Palsy

**DOI:** 10.1371/journal.pone.0223621

**Published:** 2019-10-11

**Authors:** Marina Picillo, Sofia Cuoco, Immacolata Carotenuto, Filomena Abate, Roberto Erro, Giampiero Volpe, Maria Teresa Pellecchia, Eleonora Catricalà, Stefano Cappa, Paolo Barone

**Affiliations:** 1 Center for Neurodegenerative diseases (CEMAND), Department of Medicine, Surgery and Dentistry, Neuroscience section, University of Salerno, Salerno, Italy; 2 University School for Advanced Studies IUSS Pavia, Pavia, Italy; 3 IRCCS San Giovanni di Dio Fatebenefratelli, Brescia, Italy; Anadolu University, TURKEY

## Abstract

**Background:**

Progressive Supranuclear Palsy (PSP) patients present language disturbances in tasks like naming, repetition, reading, word comprehension and semantic association compared to Parkinson’s disease (PD) and healthy controls (HC).

**Objective:**

In the present study we sought to validate a Screening for Aphasia in NeuroDegeneration (SAND) battery version specifically tailored on PSP patients and to describe language impairment in relation to PSP disease phenotype and cognitive status.

**Methods and results:**

Fifty-one PSP [23 with Richardson’s syndrome (PSP-RS), 10 with predominant parkinsonism (PSP-P) and 18 with the other variant syndromes of PSP (vPSP)], 28 PD and 30 HC were enrolled in the present study. By excluding the tasks with poor acceptability (i.e., writing and picture description tasks) and increasing the items related to the remaining tasks, we showed that the PSP-tailored SAND Global Score is an acceptable, consistent and reliable tool to screen language disturbances in PSP. However, we failed to detect major differences in language involvement according to disease phenotype. Differently, we showed that patients with dementia present worse language performances.

**Conclusions:**

Taking into account specific disease features, the combination of the SAND subscores included in the PSP-tailored SAND better represents language abilities in PSP. Furthermore, we showed that language disturbances feature PSP patients irrespective of disease phenotype, but parallels the deterioration of the global cognitive function.

## Introduction

Progressive Supranuclear Palsy (PSP) is a rare, rapidly progressive neurodegenerative disease characterized by postural instability and supranuclear vertical gaze palsy as well as by cognitive and behavioral symptoms [1]. According to the clinical diagnostic criteria proposed by the Movement Disorder Society (MDS)[2], language impairment is part of the complex spectrum of disturbances affecting patients with PSP. As such, PSP with predominant speech-language disorder (PSP-SL) is recognized as an independent clinical phenotype reaching the diagnostic level of possibility associated with a probable 4R-tauopathy pathology (i.e., either PSP or Cortico-basal Degeneration)[2]. However, evidence suggests that a wide spectrum of language deficits characterize also the remaining PSP phenotypes, including Richardson’s syndrome (PSP-RS). Recently, Burrell et al. reported that patients with PSP-RS present specific language deficits similarly to those affected by Primary Progressive Aphasia (PPA)[3]. To date, there is scant of evidence on the language profile of PSP patients diagnosed according to MDS clinical criteria [4].

The Screening for Aphasia in NeuroDegeneration (SAND) battery is a brief validated tool to detect language impairment in patients affected by neurodegenerative diseases through the assessment of different components of language [5,6]. The SAND battery is proved to detect subtle language impairment in PSP phenotypes other than PSP-SL, such as lexical-semantic level disturbances in comparison with Parkinson’s disease (PD) and healthy controls (HC)[4]. However, peculiar PSP clinical features may prevent a proper application of specific language tasks included in the SAND battery.

In the present study we aimed to validate a version of the SAND battery specifically tailored for PSP and to use it to describe language performances in PSP according to disease phenotype and cognitive status.

## Methods

### Patients

Between November 2015 and December 2018, consecutive cases of suspected PSP referred to the Center for Neurodegenerative Diseases of the University of Salerno were proposed a dedicated set of assessments including a clinical interview, a motor evaluation, extensive cognitive and behavioral testing, language evaluation and brain MRI.

For each enrolled patient the MDS proposed diagnostic flowchart was applied by two specialists for movement disorders who defined the PSP phenotypes [23 PSP-RS, 10 PSP with predominant parkinsonism (PSP-P), 9 PSP with predominant corticobasal syndrome (PSP-CBS), 4 PSP with progressive gait freezing (PSP-PGF) and 5 PSP with predominant frontal presentation (PSP-F)] according to the predominant clinical features and expressed the degree of diagnostic certainty [2,7,8]. Diagnosis as well as phenotypic attribution was verified for all patients during at least one subsequent visit. As PSP-CBS, PSP-PGF and PSP-F included a limited number of patients, those subtypes were grouped together as the other variant syndrome of PSP (vPSP = 18).

In addition, two groups of age-matched HC (N = 30) and PD (N = 28) patients were also enrolled for the present study. Exclusion criteria for enrollment of PD patients were diagnosis of dementia in accordance with MDS criteria and H&Y in on state>3. Exclusion criteria for enrollment of HC were the presence of any neurological or psychiatric conditions.

The project was performed in accordance with the ethical standards laid down in the 1964 Declaration of Helsinki. As such, the study was approved by the local Ethics Committee (Campania Sud) and each subject was included upon signature of the informed consent form.

### Clinical and cognitive evaluations

Severity of the disease was evaluated with the PSP rating scale (PSP-rs)[9].

Cognitive abilities were screened with the Mini-Mental State Examination (MMSE) and the Montreal Cognitive Assessment (MoCA). Memory domain was investigated with the immediate and delayed recall scores of the Rey auditory verbal learning test (15-RAWLT) and the Rey figure recall test. Attention-executive domain was explored through the Trail Making Test (TMT), the short version of the Stroop Interference Test, the Clock design test (CDT) and the Rey figure copying test (RCF). Visuo-spatial functions were tested with the constructional apraxia test and Benton orientation line test (BJLO) [10]. Language was explored with two sub-tests from the Neuropsychological Examination of Aphasia battery (ENPA), the non-word repetition test and the auditory comprehension test of sentences.

Functional autonomy was evaluated with the Instrumental Activities of Daily Life (IADL), while depression and apathy with the Beck Depression Inventory II (BDI-II) and Apathy Evaluation Scale (AES), respectively [11,12].

Using the z scores computed with the scores from the HC group, each PSP patient was classified as having PSP with normal cognition (PSP-NC = 4), PSP with mild cognitive impairment (MCI) single domain (PSP-MCIsd = 9), PSP with MCI-multiple domain (PSP-MCImd = 24) and PSP with dementia (PSP-D = 12) [13,14].

Due to the lack of specific MCI criteria for PSP, MDS MCI criteria for Parkinson’s disease were applied [14]. Patients presenting any type of cognitive/behavioral decline associated with impairment of IADL were considered as affected by dementia (PSP-D), according to Statistical Diagnostic Manual of Psychiatry–5th Edition (DSM-5).

### Language testing

Language was evaluated with the SAND battery [5,6]. The SAND Global Score including the 23 task-related scores was computed according with a previously described process [5]. In brief, the SAND global score is a frequency count of the pathologically impaired sub-scores with higher scores indicating more severe impairment. However, SAND Global score acceptability and consistency in PSP patients was suboptimal due to a high proportion of missing data in the writing and connected speech tasks (S1 Appendix). Therefore, following the three steps process as noted in S1 Appendix, a PSP-tailored SAND Global Score was created, reducing the impact of the writing and picture description subscores and expanding the relevance of the remaining tasks subscores (Table 1). The PSP-tailored SAND Global Score ranges from 0 to 19, with higher scores indicating greater impairment. (S1 Appendix).

**Table 1:** PSP-tailored SAND Global score (from 0 to 19).

A)Naming 1)Total 2)Living 3)Non-living B)Sentence comprehension C)Single word comprehension 1)Total 2)Living 3)Non-living D)Repetition 1)Total 2)Words 3)Non words E)Sentence repetition 1)Total 2)Predictable 3)Unpredictable F)Reading 1)Total 2)Words (regular and irregular) 3)Non words G)Semantic associations H)Writing 1)Information units I)Picture description 1)Informative units

### Statistical analysis

After checking for normality distribution with the Kolmogoroy-Smirnov test, differences in variables between groups were computed with χ^2^ or the Kruskal-Wallis tests as appropriate. Pairwise comparisons were performed with Mann-Whitney's U test.

Acceptability and internal consistency were explored for both the SAND Global Score and the PSP-tailored SAND Global Score. Acceptability was considered appropriate for each Global Score if ≤15% of the respondents totalized the lowest and highest possible scores (floor and ceiling effect) and for each Global Score item if there were ≤5% of missing values. Moreover, skewness of Global Scores (limits, -1 to +1) was determined [15].

Internal consistency was evaluated by means of Cronbach’s alpha [16]. A value≥0.70 was considered as acceptable [17]. Since the SAND Global Score showed suboptimal acceptability and consistency in PSP patients (see Results and S1 Appendix), subsequent analyses were performed only for the PSP-tailored SAND Global Score.

Scaling assumptions referring to the correct grouping of items and the appropriateness of their summed score were checked using corrected item-total correlation for PSP-tailored SAND Global Score (standard, ≥0.40 [18]).

Construct validity was explored with non-parametric Spearman’s correlation between the PSP-tailored SAND Global Score and other language testing as well as with cognitive and behavioral testing. Correlations were considered strong with coefficient>0.70 and moderate with coefficient between 0.30 and 0.70. SAND scores were not expected to correlate with memory and behavioral testing, while were expected to correlate with other language and cognitive testing.

ROC analysis was performed for the PSP-tailored SAND Global Score to identify the optimal cut off to detect language impairment in PSP patients compared to both PD and HC. Sensitivity, specificity, positive predictive value (PPV) and negative predictive value (NPV) and diagnostic accuracy in comparison to clinical diagnosis were assessed at the best threshold for classification.

Post-hoc Bonferroni test was used to correct for multiple comparisons. Statistical analysis was performed with SPSS (Version 23).

## Results

Sixty-two PSP patients were considered for the present study, but 11 were excluded according to specific inclusion/exclusion criteria detailed above. In detail, in six patients the clinical diagnosis of PSP was not confirmed in subsequent visits, four patients were not able to complete the SAND battery and one patient presented PSP-SL. The final cohort, thus, included 51 PSP patients (Table 2). According to MDS degrees of diagnostic certainty, all PSP patients had a diagnosis of probability (ie, presenting either a clear limitation of the range or decreased velocity and amplitude of vertical gaze plus other features) but those-by definition- with PSP-CBS [2]. As such, although PSP-CBS is featured by either a clear limitation of the range or decreased velocity and amplitude of vertical gaze, the presence of a corticobasal syndrome still raises the differential diagnosis with Corticobasal disease. Since no in vivo biomarkers are available differentiating PSP from Corticobasal disease, thus, PSP-CBS remains a diagnosis of possibility [2]. PSP patients presented worse performances on both the SAND Global Score and the PSP-tailored SAND Global Score compared with both PD and HC, reflecting, thus, worse language abilities (Table 2).

**Table 2:** Demographic and clinical features of enrolled subjects.

	PSP (51)	PD (28)	HC (30)	p
Age, years	71.00 (10.0)	67.00 (8.0)	66.00 (13.5)	0.229
Disease duration, years	4.00 (4.00)	5.00 (6.0)	NA	0.271
Sex, men, n (%)	29 (56.9)	21 (75)	11 (36.7)	0.013
Education, years	9.00 (9.0)	11.5 (8)	8.00 (10.5)	0.347
MMSE	24.00 (6.0)	28.00 (3.0)	27.00 (2.0)	**<0.001**^**a**^^,^^**b**^
SAND Global Score	6.00 (4.0)	2.00 (2.8)	1.50 (3.0)	**<0.001** ^**a**^^,^^**b**^
PSP-tailored SAND Global Score	8.00 (8.0)	1.00 (4.8)	1.00 (2.0)	**<0.001**^**a**^^,^^**b**^
PSP-rs	43.50 (23.8)	NA	NA	NA

Data are in median (Interquartile range, IQR), unless otherwise specified.

Significance threshold corrected for multiple comparisons = 0.002; significant differences are highlighted in bold.

Abbreviations:

a: PSP versus HC p < 0.001

b: PSP versus PD p < 0.001; HC: healthy controls; MMSE: Mini-Mental State Examination; NA: not applicable; PD: Parkinson’s disease; PSP: progressive supranuclear palsy; PSP-rs: progressive supranuclear palsy—rating scale; SAND: Screening for Aphasia in NeuroDegeneration.

### Validation phase

Although no floor effect was observed, a tendency to ceiling effect was reported for the SAND Global score (0.2% of participants obtained the lowest possible score and 15.2% the highest possible score). Skewness was 0.527. However, missing values were 30.7% and 13.3% in writing and picture description tasks, respectively, compared to 0% in the remaining tasks (S1 Appendix). Cronbach’s alpha for the SAND Global score was 0.405 and, thus, it was considered suboptimal for internal consistency [16]. Reducing the items related to the tasks with suboptimal acceptability (i.e., writing and picture description tasks) and increasing the items related to the remaining tasks significantly improved Cronbach’s alpha from 0.405 to 0.887 indicating high-level internal consistency (S1 Appendix). By removing additional items, no further improvement of Cronbach’s alpha was detected. Therefore, the 19-items PSP-tailored SAND Global Score was conceived. Neither ceiling or floor effect were observed for the PSP-tailored SAND Global Score (lowest possible score = 0, 4.8% of the participants; highest possible score = 17, 0.9% of the participants). Skewness of the PSP-tailored SAND Global Score was 0.965. All the PSP-tailored SAND Global Score items presented excellent acceptability as there were no missing data and 100% of data were computable. Scaling assumptions referring to the correct grouping of items and the appropriateness of their summed score were checked using corrected item-total correlation (standard, ≥0.40).

Spearman’s correlation confirmed convergent validity of the single tasks included in the PSP-tailored SAND Global Score, demonstrating significant moderate correlations with other language testing (Table 3). As for the other cognitive tests, moderate correlation was demonstrated with measures of global cognition as the MoCA, but not with the MMSE, as well as with tests exploring visuospatial and attention-executive domains. No correlation was shown with tests exploring memory domain or behavioral scales, while moderate correlation emerged with disease severity as assessed with the PSP-rs (S1 Appendix).

**Table 3:** Spearman’s correlation between single tasks of the PSP-tailored SAND Global Score and other language tests.

SAND Task	Language tests	Spearman’s correlation	p
Naming	CaGi naming [19]	0.523	**0.001**
Word comprehension	Auditory sentence comprehension (ENPA)	0.453	0.003
	Visual sentence comprehension (ENPA)	0.559	**0.002**
Sentence comprehension	Auditory sentence comprehension (ENPA)	0.577	**<0.001**
	Visual sentence comprehension (ENPA)	0.655	**<0.001**
Words/non words repetition	Word repetition (ENPA)	0.600	**<0.001**
	Non-word repetition (ENPA)	0.522	**<0.001**
Sentence repetition	Sentence repetition (ENPA)	0.362	0.045
Reading	Word repetition (ENPA)	0.441	0.004
	Non-word repetition (ENPA)	0.417	0.006
	Auditory sentence comprehension (ENPA)	0.616	**<0.001**
	CaGi naming [19]	0.478	0.004
	Visual sentence comprehension (ENPA)	0.482	0.011
Semantic association	CaGi naming [19]	0.500	0.003
	Auditory sentence comprehension (ENPA)	0.444	0.020
Writing I.U.	Category fluency	0.339	0.035
Connected speech I.U.	Word repetition (ENPA)	0.459	0.004
	Non-word repetition (ENPA)	0.545	**<0.001**
	CaGi naming [19]	0.626	**<0.001**

Significance threshold corrected for multiple comparisons = 0.002; significant differences are highlighted in bold.

Abbreviations: ENPA = Esame Neuropsicologico dell’Afasia; I.U = Informative Units; PSP = Progressive Supranuclear Palsy; SAND = Screening for Aphasia in NeuroDegeneration.

### Determining the optimal cut off of the PSP-tailored SAND Global Score

ROC analysis was used to assess the discriminatory power of the PSP-tailored SAND Global Score in identifying language impairment in PSP compared to both HC and PD.

As for the comparison with HC, the ROC analysis showed an 87.6% discriminatory power [95% confidence interval (CI), 80.1–95.2%]. The determined optimal cut off was 3 showing 74.5% sensitivity, 80% specificity, 86.4% positive predictive value (PPV), 64.9% negative predictive value (NPV) and 76.5% diagnostic accuracy (S1 Appendix).

As for the comparison with PD, the ROC analysis showed an 80% discriminatory power (95%CI, 69.7–91.2%). The determined optimal cut off was 3 showing 74.5% sensitivity, 71.4% specificity, 82.6% PPV, 60.6% NPV and 73.4% diagnostic accuracy (S1 Appendix).

### PSP-tailored SAND and disease features

SAND task subscores in PSP, PD and HC are shown in S1 Appendix. PSP patients reported worse outcome in all SAND task subscores as well as in the PSP-tailored SAND Global Score compared to both PD and HC.

No differences were detected in the PSP-tailored SAND Global Score among patients with different disease phenotypes (Table 4).

**Table 4:** PSP-tailored SAND in PSP disease phenotypes.

	Continuous scores				Impaired scores (%)			
SAND task	PSP-RS (23)	PSP-P (10)	vPSP (18)	p	PSP-RS (23)	PSP-P (10)	vPSP (18)	p
*Naming*								
Total	10 (4.25)	13 (6)	10 (5.25)	0.348	47.8	30	38.9	0.614
Living	6 (3.5)	6 (3)	4 (4)	0.857	34.8	20	33.3	0.685
Non-living	5 (2)	7 (1)	5 (1.75)	0.072	65.2	20	61.1	0.045
*Sentence comprehension*	6 (3.5)	8 (1)	7 (3)	0.378	50	40	50	0.852
*Single word comprehension*								
Total	10 (3)	12 (1)	12 (2)	0.047	60.9	10	44.4	0.026
Living	5 (3)	6 (1)	6 (1.5)	0.046	60.9	20	44.4	0.093
Non-living	5 (2)	6 (1)	6 (1)	0.444	39.1	10	16.7	0.119
*Repetition*								
Total	7 (3.5)	8 (4)	6 (2)	0.331	34.8	50	58.8	0.308
Words	6 (1.5)	6 (1)	5 (2)	0.448	21.7	20	33.3	0.634
Non words	1 (2)	2 (3)	1 (2.5)	0.752	26.1	50	44.4	0.313
*Sentence repetion*								
Total	3 (2.5)	3 (1)	2 (4)	0.491	43.5	40	61.1	0.436
Predictable	1 (1.5)	1 (1)	1 (2)	0.763	52.2	60	61.1	0.827
Unpredictable	1 (1)	1 (1)	1 (1.5)	0.316	17.4	10	38.9	0.145
*Reading*								
Total	14 (6.5)	14 (4)	15 (8.5)	0.835	47.8	40	50	0.875
Words	11 (4.5)	10 (4)	11 (5)	0.949	47.8	60	50	0.809
Non words	3 (2)	4 (1)	3 (3)	0.081	43.5	0	38.9	0.043
*Semantic associations*	2 (1.5)	2 (3)	2 (1)	0.677	13	20	16.7	0.871
*Writing*								
Information Units	3 (2.5)	4 (1)	2 (4)	0.030	25	0	42.9	0.088
*Picture description*								
Information Units	4 (2.5)	3 (7)	4 (2)	0.625	42.1	50	38.9	0.869
**PSP-tailored SAND Global Score**	9 (9.5)	4 (7)	7 (5.5)	0.364	69.6	70	83.3	0.565

Significance threshold corrected for multiple comparisons < 0.001.

PSP-D showed worse PSP-tailored SAND Global Score compared to both PSP-MCIsd and PSP-NC. However results were not significant when correcting for multiple comparisons (Table 5).

**Table 5:** PSP-tailored SAND in PSP according to cognitive status.

	Continuous scores					Impaired scores (%)				
SAND task	PSP-D (12)	PSP-MCImd (24)	PSP-MCI sd (9)	PSP-NC (4)	p	PSP-D (12)	PSP-MCImd (24)	PSP-MCIsd (9)	PSP-NC (4)	p
*Naming*										
Total	10 (5)	10 (5)	11.5 (5.25)	14 (0)	0.094	58.3%	45.8%	22.2%	0%	0.209
Living	5 (2.75)	5 (4)	5 (3.25)	7 (0)	0.303	33.3%	33.3%	33.3%	0%	0.693
Non-living	5 (3)	5 (1.5)	6.5 (3)	7 (0)	0.065	75%	58.3%	44.4%	0%	0.118
*Sentence comprehension*	5 (3.5)	7 (3)	7.5 (1.25)	10 (4)	*0*.*019*^a^^,^^c^^,^^e^^,^^f^	66.7%	50%	25%	0%	0.055
*Single word comprehension*										
Total	10 (4)	10 (4)	11.5 (1)	12 (1)	*0*.*016*^a^^,^^c^^,^^e^^,^^f^	66.7%	45.8%	22.2%	0%	*0*.*042*^a^^,^^c^
Living	4 (3)	5 (3)	6 (1)	6 (1)	0.056	66.7%	41.7%	44.4%	0%	0.093
Non-living	5 (2)	5 (2)	6 (0.25)	6 (1)	0.175	41.7%	25%	11.1%	0%	0.327
*Repetition*										
Total	7 (3.5)	7 (3)	7.5 (2.75)	7.5 (2)	0.316	50%	50%	22.2%	66.7%	0.590
Words	6 (2)	5 (2)	6 (1)	6 (2)	*0*.*021*^d^^,^^f^	33.3%	37.5%	0%	0%	0.119
Non words	1 (2.5)	2 (2)	2 (2.5)	1 (2.5)	0.846	41.7%	41.7%	22.2%	50%	0.621
*Sentence repetion*										
Total	3 (3)	3 (2)	2 (2)	6 (0)	0.197	58.3%	50%	55.6%	0%	0.351
Predictable	1 (1)	1 (2)	1 (0.5)	3 (0)	0.233	66.7%	62.5%	55.6%	0%	0.192
Unpredictable	1 (1)	1 (1)	1 (1.5)	3 (0)	0.102	41.7%	16.7%	22.2%	0%	0.304
*Reading*										
Total	11 (8)	14 (4)	16 (3.25)	15 (0)	0.039^a^	66.7%	50%	11.1%	25%	*0*.*047*^a^^,^^d^
Words	9 (6.5)	11 (4)	12 (2.25)	11 (0)	0.140	66.7%	50%	33.3%	25%	0.256
Non words	2 (2.5)	4 (2)	4 (1)	4 (0)	*0*.*029*^a^^,^^c^	58.3%	29.2%	11.1%	0%	*0*.*021*^a^^,^^c^
*Semantic associations*	2 (1.5)	2 (1)	2.5 (1.5)	3 (0)	0.355	25%	16.7%	11.1%	0%	0.724
*Writing*										
Information Units	1 (3)	3 (3)	4 (1.5)	4 (0)	*0*.*009* ^a^^,^^b^^,^^c^	66.7%	19%	0%	*25%*	*0*.*012*^*a*^^,^^*b*^
*Picture description*										
Information Units	3 (3.5)	4 (3)	4 (4)	3 (0)	0.475	63.6%	33.3%	28.6%	50%	0.488
**PSP-tailored SAND Global Score**	9 (10)	8 (9)	5 (6.5)	3 (0)	*0*.*024*^a^^,^^c^	83.3%	75%	66.7%	50%	0.603

No significant differences were detected according with the significance threshold corrected for multiple comparisons (< 0.001). However, in Italics are highlighted significant differences detected with the significance threshold set at p < 0.05

a = Dementia vs MCI-sd

b = Dementia vs MCI-md

c = Dementia vs NC

d = MCI-sd vs MCI-md

e = MCI- sd vs NC

f = MCI-md vs NC.

## Discussion

The present study showed that the PSP-tailored SAND battery is acceptable, reliable, and easily applicable to PSP patients. By removing subscores with high proportion of missing values and expanding subscores of the remaining tasks, we used the best combination of SAND tasks to screen language ability in PSP leading to a significant improvement in consistency and acceptability as compared to the original SAND Global Score [5,15]. As a matter of fact, differently from patients with PPA, PSP patients disclose peculiar clinical features possibly impacting performances on specific language tasks. Specifically, ocular movement abnormalities may hamper the visual exploration of the picture description and possibly impact the performances of connected speech task for non linguistic reasons. Similarly, the writing task can be affected by both apraxia and bradykinesia. The combination of SAND tasks included in the PSP-tailored SAND Global Score overcome such limits showing high acceptability since data were computable for 100% and the percentage of missing values was 0% for all items. The excellent acceptability by PSP patients is also supported by the absence of both ceiling and floor effects as well as by the optimal skewness.

Furthermore, the internal consistency of the PSP-tailored SAND battery is high and acceptable (Cronbach’s alpha = 0.887; item—total score correlation≥0.40 for all items) suggesting a coherent representation of all the language functions screened. As for convergent construct validity, each task of the PSP-tailored SAND battery showed significant moderate correlation values with other corresponding language testing. Furthermore, the PSP-tailored SAND Global Score showed moderate correlation with measures of global cognition as well as with cognitive tests exploring attention-executive and visuospatial domains. The positive association with the PSP-rs suggests a correlation between language abilities and severity of disease. No association was shown with behavioral assessments suggesting divergent validity between language function and apathy and depression burden in PSP patients.

As for the discriminatory power of the PSP-tailored SAND Global Score, the optimal cut off of 3 demonstrated an adequate sensitivity and specificity profile in identifying language impairment compared to both PD and HC. This is the first study showing a cut off for a language battery differentiating PSP from PD and HC. Previous evidence showed the SAND cut off of 5 was able in differentiating PPA from patients affected by movement disorders (PD and PSP)[5].

Confirming previous findings on a smaller cohort of patients [6], PSP patients other than PSP-SL present language disturbances when compared to both PD and HC age-matched groups (S1 Appendix).

As for language evaluation according to disease phenotype, we failed to detect significant differences suggesting language is globally involved in PSP irrespective of the specific phenotype. Confirming previous findings [13], available clinical and cognitive assessments hardly capture clinical differences among MDS PSP phenotypes.

As for the relationship between language and cognitive status, we detected a trend for worse language performances in PSP-D compared to both PSP-MCIsd and PSP-NC suggesting that language deficit may be related to the extent of impairment of the cognitive networks.

Our study has several strenghts. Firstly, the large sample size of early PSP patients enrolled (median disease duration = 4 years) representing the different phenotypes of the disease as well as the inclusion of age-matched groups of PD and HC subjects. Secondly, all included patients underwent a thorough evaluation with an extensive battery of clinical assessments by a specialist for movement disorder in a third level center and were diagnosed according to recent MDS criteria [2]. Finally, we are the first to propose an evaluation of language abilities in PSP taking into account specific disease features possibly impacting on language evaluation.

On the other hand, we acknowledge the lack of pathological confirmation, still the gold standard for PSP diagnosis, is a major limitation of our study. Another limitation of our study is the lack of cross-validation procedures for the ROC analysis which can lead to an under- or over-estimation of the PSP-tailored SAND Global score cut-off to discriminate between PSP and PD and HC subjects. However, as ocular disturbances and postural instability remain the cardinal features of PSP, language testing, as the SAND battery, would not represent a diagnostic testing for such condition. Finally, we missed to evaluate the motor speech component with appropriate instruments.

In conclusion, the combination of the SAND subscores included in the PSP-tailored SAND Global Score represents an acceptable and reliable tool to screen for language abilities in PSP. Furthermore, we showed that language disturbances feature PSP patients irrespective of disease phenotype, but may parallel the deterioration of the global cognitive function.

## Supporting information

S1 Appendix Supplemental material online. (DOCX)

S2 Appendix Supplemental material online. (XLSX)
