# Supplementary material for: Clinical use of SAND battery to evaluate language in patients with Progressive Supranuclear Palsy
Source: PLoS One. 2019 Oct 11;14(10):e0223621. doi: 10.1371/journal.pone.0223621 (PMC6788681; doi:10.1371/journal.pone.0223621)
Supplement: S1 Appendix — (DOCX) [file pone.0223621.s001.docx]

**Supplemental methods**

The SAND provides a brief comprehensive language assessment tailored for patients with PPA, including: (1) Picture naming: The subject is asked to name 14 black and white object drawings; (2) Sentence comprehension: The subject is asked to choose which of two pictures matches the meaning of the sentence read by the examiner. The sentences included two short active, two short passive, two coordinates and two embedded structures; (3) Word comprehension: The subject is asked to point at the target among four object pictures in response to a spoken word; (4) Repetition: The subject is asked to repeat words and non-words read by the examiner; (5) Sentence repetition: The subject is asked to repeat the sentences read by the examiner; (6) Reading: The subject is asked to read regular and irregular words and non-words; (7) Semantic association: the subject is asked to point at the two semantically related images out of three; (8) Writing: The subject is asked to describe how to brush their teeth; (9) Picture description task: The subject is asked to describe a complex picture [5,6].

The entire battery is short and can be administered to PSP patients in less than 20 minutes [4]. For each of the 9 subtests a score can be computed. In addition, picture description and written description analysis yields additional subscores, resulting in a total of 23 task-related scores [5,6]. Hence, the SAND Global Score including the 23 task-related scores was computed according with a previously described three steps process: (1) The raw scores were adjusted by adding or subtracting the influence of age, sex, and education and corrected using available normative data [6]; (2) Corrected scores were compared with the corresponding cutoff values obtained from HC; (3) The sum of the twenty-three dichotomous variables (1=pathological, 0=normal) represented the SAND Global Score, with higher scores indicating more severe impairment (SAND global score range:0–23)[5].

**SAND Global score (0-23) (Battista 2018)**

A)Naming

1)Total

B)Sentence comprehension

C)Single word comprehension

1)Total

D)Repetition

1)Total

E)Sentence repetition

1)Total

F)Reading

1)Total

G)Semantic associations

H)Writing

1)Information units

2)Total words

3)Nouns/total words

4)Verbs/total words

5)sentences

6)Orthographic errors

7)semantic errors

I)Picture description

1)Informative units

2)Number of words

3)Nouns/words

4)Verbs/words

5)Repaired sequences/number of words

6)Sentences

7)Subordinate/sentences

8)Phonological errors/number of words

9)Semantic errors/number of words

SAND Global score acceptability and consistency in PSP patients was suboptimal due to a high proportion of missing data in the writing and connected speech tasks. More than half of PSP patients with missing data refused to complete such tasks because they felt unable to perform the assignments. As for the picture description task, this was likely due to the gaze palsy. While for the writing task, this was likely due to both apraxia and bradykinesia.

Table: Valid and missing data of SAND battery: comparison between the writing and the picture description tasks and all the remaining SAND tasks.

|  | Writing task | Picture description task | All the remaining SAND tasks |
| --- | --- | --- | --- |
| Valid data | 39 | 45 | 51 |
| Missing data | 12 | 6 | 0 |

p < 0.001 for comparison between writing task and all the other SAND tasks

p < 0.001 for comparison between picture description task and all the other SAND tasks

Therefore, following the three steps process as noted above, a PSP-tailored SAND Global Score was created, reducing the impact of the writing and picture description subscores and expanding the relevance of the remaining tasks subscores. The PSP-tailored SAND Global Score ranges from 0 to 19, with higher scores indicating greater impairment.

**PSP-tailored SAND Global score (our proposal)**

A)Naming

1)Total

2)Living

3)Non-living

B)Sentence comprehension

C)Single word comprehension

1)Total

2)Living

3)Non-living

D)Repetition

1)Total

2)Words

3)Non words

E)Sentence repetition

1)Total

2)Predictable

3)Unpredictable

F)Reading

1)Total

2)Words (regular and irregular)

3)Non words

G)Semantic associations

H)Writing

1)Information units

I)Picture description

1)Informative units

By reducing the items of the picture description and writing tasks and expanding the items of other tasks, acceptability of the SAND battery presented a significant improvement (see Results).

Additional inclusion criteria for the present study were: (a) Italian native speaker status; (b) sufficiently intelligible speech such that the intended target could be determined for the majority of words; (c) intact or corrected auditory and visual functions; (d) disease duration less than 10 years; (e) successful completion of the language testing. Additional exclusion criteria included: (a) Mini-Mental State Examination (MMSE) < 10 [5]; (b) fulfillment of the criteria for PSP-SL [2].

**Supplemental Figure. a** Summary of the diagnostic accuracy of the SAND battery for the comparison of PSP patients versus HC. **b** ROC curve for the Global Score of the SAND battery to detect patients with language dysfunction evacuate in the sample of PSP patients versus HC. **c** Summary of the diagnostic accuracy of the SAND battery for the comparison of PD patients versus PSP patients. **d** ROC curve for the Global Score of the SAND battery to detect patients with language dysfunction evaluated in the comparison of PSP patients versus PD patients.


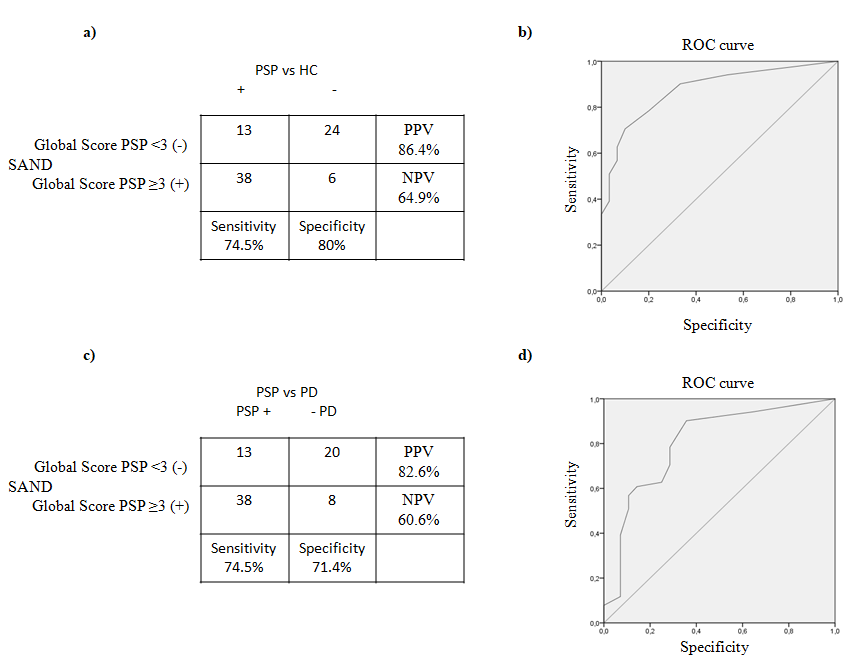


Abbreviations: HC: healthy controls; NPV: negative predictive value; PD: Parkinson’s disease; PPV: positive predictive value; PSP: progressive supranuclear palsy; ROC: receiver operating characteristic; SAND: Screening for Aphasia in NeuroDegeneration.

**Supplemental table. Spearman’s correlation between the PSP-tailored SAND Global Score and non-language tests.**

|  | **Spearman’s correlation** | **p** |
| --- | --- | --- |
| *Screening of global cognition* | | |
| MMSE | -0.058 | **<0.001** |
| MoCA | -0.564 | **<0.001** |
| *Memory domain* | | |
| RAWLT immediate | -0.296 | 0.044 |
| RAWLT recall | -0.175 | 0.238 |
| RCF recall | -0.151 | 0.345 |
| *Attention-executive domain* | | |
| CDT | -0.438 | 0.005 |
| RCF copy | -0.521 | **<0.001** |
| TMT-A | 0.645 | **<0.001** |
| Stroop color word test | 0.540 | **<0.001** |
| *Visuo-spatial domain* | | |
| Constructional apraxia | -0.405 | 0.008 |
| BJLO | -0.645 | **<0.001** |
| *Behavioral scales* | | |
| BDI-II | 0.106 | 0.511 |
| AES | 0.028 | 0.851 |
| *Disease severity* | | |
| PSP-rs | 0.501 | **<0.001** |

Significance threshold corrected for multiple comparisons = 0.003; significant differences are highlighted in bold.

Abbreviations: AES: Apathy Evaluation Scale; BDI-II: Beck Depression Inventory II; BJLO: Benton’s Judgment of Line Orientation; CDT: Clock Drawing test; MMSE: Mini-Mental State Examinaton; MoCA: Montreal Cognitive Assessment battery; PSP-rs: Progressive Supranuclear Palsy – rating scale; RAVLT: Rey’s auditory 15-word learning test; RCF: Rey figure test; TMT-A: Trial Making Test A.

**Supplemental table. PSP-tailored SAND in PSP, PD and HC**

|  | **Continuous scores** | | | | **Impaired scores (%)** | | | |
| --- | --- | --- | --- | --- | --- | --- | --- | --- |
| **SAND task** | **PSP (51)** | **PD (28)** | **HC (30)** | **p** | **PSP (51)** | **PD (28)** | **HC (30)** | **p** |
| *Naming* | | | | | | | | |
| Total | 10 (5) | 13 (2) | 13 (2) | **<0.001^a,b^** | 41.2 | 10.7 | 6.7 | **<0.001^a,b^** |
| Living | 5 (4) | 7 (1.8) | 7 (1) | **<0.001 ^a,b^** | 31.4 | 7.1 | 0 | **<0.001 ^a,b^** |
| Non-living | 6 (2.2) | 6.75 (1) | 7 (1.5) | **<0.001 ^a,b^** | 54.9 | 7.1 | 23.3 | **<0.001 ^a,b^** |
| *Sentence comprehension* | 7 (3) | 8 (0) | 8 (1) | **<0.001 ^a,b^** | 49 | 10.7 | 10 | **<0.001 ^a,b^** |
| *Single word comprehension* |  |  |  |  |  |  |  |  |
| Total | 11 (3.5) | 12 (0) | 12 (1) | **<0.001 ^a,b^** | 45.1 | 10.7 | 6.7 | **<0.001 ^a,b^** |
| Living | 6 (2) | 6 (0) | 6 (0) | **<0.001 ^a,b^** | 47.1 | 14.3 | 6.7 | **<0.001 ^a,b^** |
| Non-living | 6 (1) | 6 (1) | 6 (1) | **<0.001 ^a,b^** | 25.5 | 7.1 | 3.3 | 0.011 |
| *Repetition* | | | | | | | | |
| Total | 7 (3) | 8 (2) | 9 (2) | **<0.001 ^a,b^** | 74.2 | 21.4 | 6.7 | **<0.001 ^a,b^** |
| Words | 6 (1.5) | 6 (0) | 6 (0) | **<0.001 ^a,b^** | 25.5 | 0 | 7.1 | 0.003 |
| Non words | 2 (3) | 2 (2) | 3(2) | **<0.001 ^a,b^** | 37.3 | 14.3 | 10 | 0.008 |
| *Sentence repetion* | | | | | | | | |
| Total | 3 (2.5) | 4.5 (3) | 5 (2) | **<0.001 ^a,b^** | 49 | 21.4 | 3.3 | **<0.001 ^a,c^** |
| Predictable | 1 (1) | 2 (2) | 2 (1) | **<0.001 ^a,b,c^** | 56.9 | 32.1 | 13.3 | **<0.001 ^a,b^** |
| Unpredictable | 1 (1) | 2 (2) | 2 (1) | **<0.001 ^a,b^** | 23.5 | 7.1 | 0 | 0.005 |
| *Reading* | | | | | | | | |
| Total | 14 (6) | 15 (2) | 16 (1) | **<0.001 ^a,b^** | 47.1 | 10.7 | 13.3 | **<0.001 ^a,b^** |
| Words | 11 (4) | 12 (1) | 12 (0.5) | **<0.001 ^a,b^** | 51 | 17.9 | 16.7 | 0.001 |
| Nonwords | 4 (2) | 4 (1) | 4 (0) | 0.003 | 33.3 | 10.7 | 3.3 | 0.002 |
| *Semantic associations* | 2 (1) | 3 (1) | 3 (2) | 0.002 | 15.7 | 0 | 6.7 | 0.059 |
| *Writing* | | | | | | | | |
| Information Units | 3 (3) | 4 (3) | 6 (1) | 0.001 | 26.2 | 25 | 0 | 0.009 |
| *Picture description* | | | | | | | | |
| Information Units | 4 (2.5) | 6 (3.75) | 5 (3) | 0.002 | 42.2 | 25 | 13.3 | 0.022 |
| **PSP-tailored SAND Global Score** | 8 (8) | 1 (4.75) | 1 (2) | **<0.001 ^a,b^** | 74.5 | 28.6 | 20 | **<0.001 ^a,b^** |

Significance threshold corrected for multiple comparisons < 0.001; significant differences are highlighted in bold.

^a^= PSP vs HC p<0.05

^b^= PSP vs PD p<0.05

^c^= HC vs PD p<0.05
